# Cytokine release syndrome induced by pembrolizumab: A case report

**DOI:** 10.1097/MD.0000000000031998

**Published:** 2022-12-09

**Authors:** Xinyu Zhang, Zhibin Fu, Chaoguang Yan

**Affiliations:** a Shandong University of Traditional Chinese Medicine, China; b Weifang Hospital of Traditional Chinese Medicine, China.

**Keywords:** cytokine release syndrome, immune checkpoint inhibitors, immune-related adverse events, pabrolizumab

## Abstract

Cytokine release syndrome (CRS) is an immune hyperactivation phenomenon in immunotherapy and, unlike other immune-related adverse events, only a few case reports have documented CRS due to the use of immune checkpoint inhibitors. In this article, we report a case of 2 episodes of CRS and delirium caused by pembrolizumab in a short period of time. This helps clinicians to understand CRS and to improve the diagnosis and treatment of immune-related adverse events.

**Patient concerns:**

A 67-year-old patient with lung cancer developed fever, delirium, acute renal insufficiency, and acute cardiac insufficiency after 9 cycles of pablizumab therapy, and reappeared with these symptoms 1 week after improvement with glucocorticoid therapy.

**Diagnoses:**

The patient presented with concomitant cardiac insufficiency, hepatic and renal failure, delirium with high C-reactive protein levels and the patient’s response to glucocorticoids, and exclusion of cerebrovascular accident and severe infection, resulting in a final diagnosis of CRS.

**Interventions:**

Glucocorticoid therapy and symptomatic support treatment.

**Outcomes:**

After 2 hospitalizations, the patient did not develop CRS.

**Lessons:**

To our knowledge, this is the first case of delirium and CRS that occurred twice in a short period of time. This patient had no immune-related adverse reactions during the previous 9 immunotherapy sessions. This adverse reaction occurred after the inflammation of the wisdom teeth and was presumed to be related to an overstimulation of the immune response due to infection. Premature discontinuation of hormones for the patient’s 1st treatment of CRS may be the reason for the 2nd occurrence of CRS. Therefore, timely and full course of glucocorticosteroids is a key therapeutic measure to cause CRS after the use of immune checkpoint inhibitors.

## 1. Introduction

Immune checkpoint inhibitors (ICIs) are an effective treatment for many cancers, however, ICIs inevitably produce immune-related adverse events (irAEs).^[1]^ Cytokine release syndrome (CRS) is a form of immune hyperactivation in immunotherapy. Patients with CRS can develop fever, hypotension and multi-organ dysfunction, which can be life-threatening.^[2]^ Unlike other irAEs, only a few case reports have documented ICIs as pharmacological triggers of CRS in cancer patients.^[3–9]^ In this article, we report on a 67-year-old male patient with lung cancer. The patient experienced CRS twice in a short period of time after 9 cycles of pablizumab immunotherapy.

## 2. Case presentation

A 67-year-old man presented with a cough in January 2021. The patient had been smoking for 50 years. The patient’s pathology showed squamous carcinoma by percutaneous biopsy under CT. The diagnosis of stage IV squamous carcinoma of the upper lobe of the right lung cT2N3M1 (liver) was later perfected. Genetic testing showed that all lung cancer driver genes were negative. The “paclitaxel + carboplatin + pablizumab” regimen was administered for 6 weeks from January 23 to May 30, and the curative effect was partial remission. Pabrolizumab monotherapy maintenance was administered for 3 cycles from July 2 to September 6, during which the lung lesions were unchanged on review and the liver lesions continued to shrink, with partial remission efficacy. On September 25, the patient was admitted to the hospital with inflammation of the wisdom teeth, which severely affected eating. On the night of September 29, fever appeared with a maximum temperature of 38.5°C, and the temperature returned to normal after the administration of ibuprofen. On September 30, his condition deteriorated with drowsiness, delirium, inability to answer questions accurately and cooperate with physical examination, body temperature around 38°C, ECG monitoring showed: heart rate 150 to 160 bpm, BP 87/59 mm Hg, SPO2 91% to 93% under oxygenation. Cranial and thoracic CT did not show clear metastases and cerebral hemorrhage or large infarcts. Laboratory tests showed elevated WBC, elevated Cr, elevated CK, and elevated high sensitivity C-reactive protein (hsCRP), the results of which are shown in Table 1. Consider co-infection, acute renal insufficiency, and myocarditis. Meropenem 1 g tid anti-infection and methylprednisolone 80 mg bid were administered. From October 2, the body temperature was normal, the renal function and myocardial enzymes were better than before, and the blood pressure and heart rhythm were back to normal. On October 4, the patient’s consciousness gradually recovered, the infection was controlled, Cr decreased, CK decreased, and hsCRP decreased, as shown in Table 1. Methylprednisolone was reduced to 60 mg bid, and antibiotics were stopped. On October 7 blood routine, biochemistry and cardiac enzyme profile were normal, and methylprednisolone was reduced to 40 mg bid. The patient was discontinued from hormone therapy and discharged to rest on October 10 after her general condition improved.

**Table 1:** Laboratory test results.

	Sept. 30	Oct. 2	Oct. 4	Oct. 7	Oct. 25	Oct. 27	Oct. 28	Oct. 30	Nov. 5	Normal range
Blood routine										
White blood cell (WBC)	12.1		10.9	8.2	15.5			10.3	7.3	4–10 (*109/L)
Neutrophil percentage	72.60%		70.30%	69.20%	63.40%			68.00%	68.50%	50%–70%
Biochemistry										
Creatinine (Cr)	348	258	122	132	335	187	186	173	118	88.4–176.8 (μmol/L
Alanine aminotransferase (ALT)	18.6	19.8	19.3	22.4	38.7	387.5	783.6	326.1	20.8	5–40 (U/L)
Aspartate aminotransferase (AST)	22.4	22.9	21.9	25.5	73.2	1874.9	543.2	87.5	23.2	8–40 (U/L)
Myocardial enzymes										
Creatine phosphokinase (CK)	334	304	282	182	358	1934	1564	732	173	50–310 (U/L)
Creatine kinaseisoenzyme (CK-MB)	22.8	17.3	18.4	18.2	87.2	65.7	55.9	46.2	14.7	0–25 U/L
Myoglobin (Mb)	58	53	55	49	732	744	683	531	47	28–72 (ng/mL)
Others										
Brain natriuretic peptide (BNP)	32.21	31.54	33.52	31.48	29.66	348.09	339.07	298.51	40.53	<100 pg/mL
High sensitivity C-reactive protein (hsCRP)	223.67	153.47	43.59	7.82	83.58	79.21	74.23	71.95	7.68	0.068–8.2 mg/L

The patient developed a fever with no apparent cause from October 18, with a maximum temperature of 38°C, accompanied by significant malaise, chest tightness, dyspnea and less urination. He was admitted to the hospital on October 25. Laboratory tests showed elevated WBC, elevated Cr, elevated CK, elevated CK-MB, elevated Mb, and elevated hsCRP. The specific results are shown in Table 1. Blood gas analysis suggests severe metabolic acidosis with hyperlactatemia. He was treated with rehydration, continuous renal replacement therapy, meropenem anti-infection, and methylprednisolone 80 mg bid. The patient developed delirium on October 27. Blood gas analysis indicates metabolic acidosis and hyperlactatemia is significantly better than before. A repeat cardiac enzyme profile showed further elevation of CK, elevation of BNP, and elevation of ALT and AST, as shown in Table 1. Considering the patient’s acute liver failure, he was treated with polyenylphosphatidylcholine and ornithine aspartate for liver protection. On October 28, the patient’s mental status returned to normal, and the cardiac enzyme profile, Cr, ALT, and AST were all better than before. The dosage of methylprednisolone was adjusted to 40 mg bid. On October 30, he did not complain of any special discomfort and slept well at night. Myocardial enzyme profiles, AST, ALT, BNP, and hsCRP continued to improve, and the results are shown in Table 1. Discontinue hepatoprotective drugs and antimicrobials and continue hormone therapy. The patient’s liver and kidney functions and cardiac enzyme profile returned to normal on November 5. The patient was discharged from the hospital and continued oral methylprednisolone treatment for 2 months and then discontinued successfully without further adverse effects.

## 3. Discussion

In addition to surgery, chemotherapy and radiotherapy, ICIs are the next generation of drugs in cancer treatment. However, the use of ICIs is inevitably associated with irAEs, of which CRS has been reported in a few case reports. CRS is a systemic inflammatory disease mediated by several cytokines, including IL-6, TNF-α α IFN-γ, IL-2, IL-8, IL-10, and granulocyte-macrophage colony-stimulating factor (GM-CSF), of which IL-6 is considered the main protagonist.^[10]^ Mild cases of CRS are characterized by fever, while severe cases are often characterized by hypotension and multi-organ dysfunction. High C-reactive protein levels, elevated transaminases and creatinine are common abnormal laboratory indicators in patients with CRS.^[11]^ Common complications of CRS include cardiac insufficiency, adult respiratory distress syndrome, neurological toxicity, renal and hepatic failure, and disseminated intravascular coagulation.^[2]^ Meanwhile, clinical experience has shown that glucocorticoids are an effective treatment for CRS.

The patient presented twice with concurrent cardiac insufficiency, hepatic and renal impairment, delirium with high C-reactive protein levels and the patient’s response to glucocorticoids, supporting the diagnosis of CRS. At the same time, there was no significant abnormality in the cranial CT to exclude the possibility of cerebrovascular accident, and the antibacterial drugs were stopped after 5 days of anti-infection treatment without further adverse reactions, which excluded the possibility of severe infection. Although the patient’s serum cytokines were not tested, they were sufficient to diagnose the patient with CRS. The patient had no irAEs during the previous 9 immunotherapy sessions. This adverse reaction occurred after the inflammation of the wisdom teeth and was presumed to be related to an over-boosted immune response due to infection, and the second CRS may be related to reactivation of the immune system due to premature discontinuation of hormones.

The first occurrence of CRS in this patient was not clear whether it was caused by a severe infection or an adverse immune reaction, and hormone therapy was discontinued prematurely after effective treatment, and CRS occurred again 8 days after discontinuation of hormones, consistent with the memory characteristics of the immune system. The patient’s hepatic and renal insufficiency, cardiac insufficiency, and delirium all improved rapidly after treatment with hormones and anti-infection at the time of the second episode of CRS. After the hormone was gradually reduced, the patient was discharged from the hospital and continued to take oral methylprednisolone for 2 months, and no further CRS occurred after discontinuation of the drug. The shortcoming of this case was the failure to test the patient’s cytokines. Since the mechanism of CRS is inflammatory cytokines exceeding physiological levels, cytokine levels can be used as a basis for the diagnosis of CRS. Studies have shown that cytokine levels are directly proportional to the severity of CRS.^[12]^ Monitoring of cytokines in patients can help in the diagnosis of CRS and the assessment of its severity. Previous studies have shown that c reactive protein (CRP) is an acute responder to IL-6 stimulation in the liver and that CRP levels can serve as a reliable surrogate for IL-6 bioactivity.^[13]^ CRP is highly expressed in patients with CRS, and the level of CRP can predict the risk of developing severe CRS, and monitoring CRP levels during the development of CRS has clinical significance.^[2]^ In patients with CRS, CRP monitoring can replace cytokine monitoring to some extent because it is quick and inexpensive. However, it is worth noting that CRP levels are also elevated in infectious diseases and cannot be used to differentiate infectious from noninfectious inflammation. Therefore, cytokine testing should be performed in patients with suspected CRS when conditions permit.

The indications for ICIs are expanding, and with them the number of reported adverse effects is gradually increasing. Severe CRS, such as the one that occurred in this patient, can be life-threatening if left untreated. This also suggests that clinicians should fully understand the morbidity characteristics, clinical manifestations and treatment principles of CRS, and intervene in a timely manner to reduce the risk of medication use. This case also confirms the effectiveness of glucocorticoids in the treatment of CRS, and in future patients with CRS caused by pablizumab, attention should be paid to the timely and full course of glucocorticoids.

## Author contributions

**Conceptualization:** Xinyu Zhang, Chaoguang Yan.

**Data curation:** Xinyu Zhang, Chaoguang Yan.

**Funding acquisition:** Chaoguang Yan.

**Investigation:** Xinyu Zhang.

**Project administration:** Chaoguang Yan.

**Resources:** Chaoguang Yan.

**Software:** Chaoguang Yan.

**Supervision:** Zhibin Fu, Chaoguang Yan.

**Validation:** Chaoguang Yan.

**Visualization:** Xinyu Zhang.

**Writing – original draft:** Xinyu Zhang.

**Writing – review & editing:** Zhibin Fu, Chaoguang Yan.
